# β-Cell-Derived Extracellular Vesicles Boost β-Cell Functionality in Human Pancreatic Islets

**DOI:** 10.34133/bmr.0346

**Published:** 2026-03-17

**Authors:** Sarah Boucenna, Antoine Karoichan, Michael Yilma Yitayew, John V. L. Nguyen, Maryam Tabrizian

**Affiliations:** ^1^ Faculty of Dental Medicine and Oral Health Sciences, McGill University, Montreal, Quebec H3A 1G1, Canada.; ^2^ Department of Biomedical Engineering, McGill University, Montreal, Quebec H3A 2B4, Canada.

## Abstract

Extracellular vesicles (EVs) are emerging therapeutic tools in nanomedicine, yet their effects in 2-dimensional (2D) versus 3D diabetes models remain underexplored. Unlike synthetic nanoparticles, EVs’ cellular origin, innate bioactivity, and biological cargo make them attractive candidates for disease treatment. This study investigated whether β-cell-derived EVs enhance β-cell function, particularly insulin secretion. EVs were isolated from the human EndoC-βH1 β-cell line, characterized, and assessed for uptake by EndoC-βH1-derived spheroids and human donor pancreatic islets using confocal microscopy. The effect of EV uptake on spheroids and human islet function was determined through glucose-stimulated insulin secretion (GSIS) tests, enzyme-linked immunosorbent assay (ELISA), and quantitative polymerase chain reaction (qPCR) to compare insulin output and β-cell gene expression between EV-treated and untreated samples. Both spheroids and donor islets showed increased insulin production compared to controls. In spheroids, qPCR revealed elevated expression of PDX1 and SUR1. In contrast, EV-treated human islets exhibited a 3-fold increase in insulin secretion without significant changes in INS, PDX1, GCG, or SLC2A2 expression. Proteomic analysis further demonstrated enrichment in key proteins involved in β-cell function and survival in both EV-treated spheroids and islets. These findings suggest that β-cell-derived EVs can promote β-cell functionality in vitro by up-regulating key genes involved in insulin secretion. The results support the EndoC-βH1-derived spheroid model as a platform for studying human islet biology for advancing the preclinical development of EV-based therapies. This work offers new insights into the effects of β-cell-derived EVs in promoting β-cell functionality and highlights their potential to improve islet transplantation outcomes for patients with insulin-dependent type 1 diabetes.

## Introduction

Type 1 diabetes (T1D) is an autoimmune disease that affects more than 9.5 million people worldwide [1]. T1D is characterized by the destruction of pancreatic islets by the immune system, specifically by autoreactive cytotoxic T-cells, which leads to the loss of function of β-cells and thus insulin deficiency. By the time the first symptoms of T1D appear, more than 80% of β-cells have already been destroyed [2]. A curative treatment for T1D remains lacking. Current gold standards rely on exogenous insulin administration to maintain and regulate blood glucose levels; however, this approach requires repetitive insulin infusions, which can expose the patient to chronic complications and refractory hypoglycemia [3]. Given the constantly rising prevalence rates of T1D, and the major financial burdens that insulin replacement therapies present, there is an urgent need for novel therapeutic approaches capable of achieving durable, disease-modifying outcomes.

In efforts to find strategies that move beyond symptomatic management, islet transplantation has been increasingly explored as a long-term curative treatment for T1D [4,5]. Despite its promise, this approach continues to face major challenges. More than 50% of islet function is typically lost during isolation, transplantation, and the post-transplantation period, largely due to transient ischemia from loss of vascularization and extracellular matrix (ECM) degradation. This markedly reduces graft efficacy and survival, and in many cases, 2 to 3 donor pancreases are required to achieve a single successful transplantation [6]. Researchers have explored various approaches to preserve functional islet mass and thus reduce the number of pancreases needed for an islet transplantation. Examples include islet encapsulation to protect the graft from immune rejection, or co-transplantation of mesenchymal stem cells (MSCs) and regulatory T-cells (Tregs) with islets for their immunomodulatory and β-cell protective properties [7,8]. While islet encapsulation techniques provide protection, they do not specifically enhance islet functionality. On the other hand, cell therapy has its own limitations, such as heterogeneity and loss of potency over time [9]. Therefore, the exploration of targeted therapies to improve islet functionality and restore insulin production could serve as a valuable complementary approach to these existing methods, improving islet transplant survival while reducing the number of pancreases needed for an islet graft in a patient.

Extracellular vesicles (EVs) have emerged as promising candidates in this regard. EVs are nanosized particles naturally secreted by cells, carrying biologically active cargo that reflects the molecular profile of the parent cell. They have attracted significant interest over the past decade due to their innate bioactivity and reduced immunogenicity [10]. In healthy states, EVs can play important roles in supporting tissue homeostasis, while in disease contexts, they can propagate pathology to other cells and organs [11]. This makes EVs interesting tools to both better understand and treat various types of complex diseases. A large number of studies have investigated the therapeutic potential of EVs in different pathologies such as cancer, cardiovascular diseases, and musculoskeletal disorders [12,13].

Similarly, EVs have been gaining interest from the field of diabetes research and have been shown to be involved in both the physiological and pathological mechanisms of the pancreatic tissue and the immune system. For instance, Figliolini et al. [14] demonstrated the ability of islet-derived EVs to participate in endothelial crosstalk and promote β-cell function, insulin secretion, and angiogenesis. On the other hand, EVs derived from endothelial progenitor cells have been shown to promote and maintain vascularization in islets of Langerhans [15]. It has also been reported that adipose tissue macrophage (ATM)-derived EVs can induce insulin resistance when injected into healthy mice, whereas ATM-EVs from lean mice can improve glucose tolerance when administered to obese mice [16].

Given their role as insulin-producing cells, EVs derived from β-cells may also be promising tools to investigate for their potential to enhance insulin secretion in diabetes therapy. Recently, Sun et al. [17] demonstrated that administration of mouse β-cell-derived EVs could successfully improve glucose tolerance and insulin content in mouse islets, and the authors reported better immune tolerance and survival time of diabetic mice treated with the vesicles. Other groups have been able to recapitulate similar findings using human β-cell-derived EVs, indicating their valuable therapeutic potential [18]. However, the characterization of human β-cell-derived EVs and the assessment of their effect on pancreatic islet functionality remain limited and warrant further investigation to improve their potential for clinical translation in diabetes treatment.

In this work, we present a proof-of-concept study focused on exploring the potential of EVs derived from human EndoC-βH1 cells to enhance β-cell functionality and insulin secretion. Using both EndoC-βH1 cell-derived spheroids and human donor islets, a side-by-side study was performed to evaluate the effect of β-cell-derived EVs. The use of EndoC-βH-derived spheroids allowed for the assessment of EV-mediated effects on β-cell functionality in a more physiologically relevant context, as these 3-dimensional (3D) models more closely mimic primary islets compared to traditional 2D cell cultures. In both cases, the primary metric used to evaluate β-cell and islet functionality was insulin secretion. This measure includes basal and glucose-stimulated insulin secretion (GSIS), which reflects the ability of cells to respond appropriately to glucose levels and demonstrates a critical aspect of β-cell health and functionality.

To that end, β-cell-derived EVs were isolated from EndoC-βH1 conditioned media by ultracentrifugation, and their size distribution, colloidal stability, and morphology were characterized. Biocompatibility of the EVs with EndoC-βH1 spheroids and human donor islets was evaluated using alamarBlue and reactive oxygen species (ROS) assays, and their effects on insulin secretion and content were assessed by the GSIS assay at different time points. Furthermore, expression of markers relevant to β-cell functionality, including INS, PDX1, SUR1, GCG, and SLC2A2 (GLUT2), was assessed by qPCR to correlate their expression with insulin secretion after EV treatment. To provide new insights into the potential to use β-cell-derived EVs as a strategy to enhance islet functionality, bioinformatic analysis of the proteomic profiles of EV-treated EndoC-βH1 spheroids and human donor islets was also performed to determine the effect of β-cell-derived EVs on both EndoC-βH1 spheroids and human donor islets

## Materials and Methods

### β-Cell culture

EndoC-βH1 cells (Human Cell Design, Toulouse, France) were grown in serum-free Ultiβ1 medium (Human Cell Design) using flasks coated with the βCoat coating matrix (Human Cell Design) as previously described (*19*).

### Source of human pancreatic islets

Primary human pancreatic islets were purchased from Prodo Labs (Aliso Viejo, CA, USA), and the donor was a 66-year-old white male, 72″, 248 lbs, with a body mass index (BMI) of 33.7 and HbA1C of 5.9%. The donor tested negative for COVID-19 and had no known metabolic or systemic complications and no diagnosed history of diabetes or related comorbidities. The cells were 90% pure and 95% viable. After shipping, islets were retrieved and supplemented with PIM(S) culture media (Prodo Labs). The media were changed 24 h after retrieval, followed by media changes every 2 to 3 d. All experiments were conducted under ethical approval from the institutional ethics board of McGill University (institutional review board study number A02-E28-12B). Throughout the manuscript, these primary human pancreatic islets are referred to as islets.

### EndoC-βH1-derived EV isolation and characterization

EndoC-βH1 cells were cultured to a density of ~2 × 10^6^ cells/ml, washed thoroughly with phosphate-buffered saline (PBS), and transferred to serum-free media for EV collection. Conditioned media were collected every other day up to 3 times, pooled, and cleared of cells and debris by sequential centrifugation at 500*g* for 10 min and 2,000*g* for 20 min. The supernatant was filtered through 0.22-μm polyethersulfone (PES) filters and concentrated using tangential flow filtration with a 100-kDa molecular weight cutoff membrane. EVs were then isolated by ultracentrifugation at 120,000*g* for 70 min at 4 °C, washed in PBS at 150,000*g* for 70 min, and resuspended in 300 μl of PBS.

EV colloidal characterization included measurement of hydrodynamic size distribution and particle concentration by nanoparticle tracking analysis with laser type Blue488 (NanoSight NS300, Malvern Panalytical, Malvern, UK) and zeta potential analysis by the ZetaPALS analyzer (Brookhaven Instruments, Holtsville, NY, USA). Morphology was confirmed by negative-stain transmission electron microscopy using a Tecnai G2 Spirit 120 kV transmission electron microscope (TEM) (FEI Company, Hillsboro, OR, USA). Throughout the manuscript, these EndoC-βH1 human cell line-derived EVs are referred to as EVs.

### Protein quantification and Western blotting

Pierce Rapid Gold BCA Protein Assay Kit (Thermo Fisher Scientific, Waltham, MA, USA) was used for protein quantification. EV proteins (20 μg) or whole-cell lysate was loaded onto the wells of TGX Stain-Free precast gels for polyacrylamide gel electrophoresis (Bio-Rad, Hercules, CA, USA) at 120 V for 1 h before being transferred to a polyvinylidene difluoride membrane (Bio-Rad) overnight at 4 °C with current held constant at 10 mA. Afterward, the membrane was blocked using 5% milk for 1 h and probed with primary antibodies against the EV marker TSG101 (1:1,000) (BioLegend, San Diego, CA, USA) overnight at 4 °C. The membrane was then incubated with horseradish peroxidase (HRP)-conjugated secondary antibodies (1:2,000) (BioLegend) at 4 °C for 1 h, incubated with substrate solution (Thermo Fisher Scientific), and imaged using the ChemiDoc imaging system (Bio-Rad) to visualize the protein bands.

### EndoC-βH1-derived spheroid preparation

EndoC-βH1 cells were seeded into nontreated 6-well plates (Thermo Fisher Scientific) at a density of 1 × 10^6^ cells per well. The plates were placed on a shaker (80 to 90 rpm) and incubated at 37 °C and 5% CO_2_ for 5 d to promote spheroid formation. This method results in the formation of 100 to 150 spheroids per well with a size of 150 to 350 μm per spheroid. Each spheroid consists of 6,000 to 10,000 cells. Spheroid size was measured using a Zeiss Axiovert 3 brightfield microscope (Zeiss, Oberkochen, Germany) and analyzed using ImageJ software. Prior to the experiment, spheroids that are similar in shape and size are counted, selected, and transferred to appropriate wells using a micropipette and a light microscope in a Biological Safety Cabinet (BSC). Throughout the manuscript, these EndoC-βH1 human cell line-derived spheroids are referred to as spheroids.

### Assessment of EV uptake by EndoC-βH1 spheroids and human islets

EVs were labeled with PKH67 green fluorescent dye (MilliporeSigma, Burlington, MA, USA) as previously described [19]. Briefly, 50 μl of EVs was suspended in 125 μl of diluent C and 4 μl of PKH67 and incubated for 4 min at room temperature. To quench any unbound dye, 500 μl of 5% bovine serum albumin (BSA) was added before ultracentrifugation at 120,000*g* for 70 min at 4 °C. The labeled EVs were then washed with PBS under similar ultracentrifugation conditions and resuspended in PBS.

For the EV uptake assay, EndoC-βH1 spheroids and human islets were incubated with the PKH67-labeled EVs for 24 h at 37 °C and 5% CO_2_. Spheroids incubated with PKH67 in particle-free PBS were used as a control to ensure that detected signals are coming from successfully stained EVs rather than residual free dye. They were then transferred to glass-bottom Petri dishes (Cellvis, Mountain View, CA, USA) and washed thoroughly with PBS before fixing with 4% paraformaldehyde for 20 min at room temperature. After washing again 3 times with PBS and staining with Hoechst dye (1:2,000) (Thermo Fisher Scientific), the samples were imaged using an LSM 710 confocal microscope (Zeiss, Oberkochen, Germany). Mean fluorescence intensity of PKH67 referred to as MFI was quantified using the ImageJ software. The analysis was done on 3 different spheroids/islets for each condition, and the results were normalized using the corresponding Hoechst staining MFI.

### Assessing the cytotoxic effect of EVs on EndoC-βH1-derived spheroid and human islets

EndoC-βH1 spheroids and donor islets were incubated with culture media supplemented with 20 or 40 μg/ml of EVs, which after correlation with the nanoparticle track analysis (NTA) results correspond to 1.18 × 10^9^ and 2.36 × 10^9^ EV particles, respectively. To evaluate any cytotoxic effects, the metabolic activity of the cells was monitored at 24, 48, and 72 h of EV exposure using an alamarBlue assay (Thermo Fisher Scientific) per the manufacturer’s protocol.

The potential effect of the EVs on cellular oxidative stress was also assessed using an ROS assay kit (Abcam, Cambridge, UK). Following 24 h of EV treatment at 20 or 40 μg/ml, EndoC-βH1 spheroids and donor islets were incubated with dichlorodihydrofluorescein diacetate (DCFDA) for 45 min and then exposed to *tert*-butyl hydroperoxide (TBHP) for 5 h before fluorescence was measured at 485/535 nm using the SpectraMax i3x microplate reader (Molecular Devices, San Jose, CA, USA).

### Glucose-stimulated insulin secretion

EndoC-βH1 spheroids and human islets were treated with 25 μg/ml of EVs for 6, 12, and 24 h. To evaluate the impact of EV treatment on β-cell functionality, GSIS assays were performed using untreated groups as controls. Specifically, 3 samples of 100 spheroids and 100 islets each independently treated and untreated were used for the experiment. For EndoC-βH1 spheroids, assays were carried out following a 24-h glucose starvation period, whereas human islets underwent a 2-h starvation period. Samples were then incubated in 0.1% BSA Krebs buffer at 37 °C in 5% CO_2_ for 1 h, after which they were incubated for an additional 40 min under basal and stimulatory glucose conditions (0 or 20 mM glucose for spheroids; 2.8 or 28 mM glucose for islets). The starvation period and glucose concentrations for stimulation were chosen according to the manufacturer’s protocol for EndoC-βH1 cells and a previously established GSIS protocol for human islets [19–22]. Following incubation, the buffers were centrifuged at 700*g* for 5 min at 4 °C, and the resulting supernatants were collected for subsequent analysis.

Insulin concentrations in supernatants and cell lysates were quantified using an insulin ELISA kit (Mercodia, Uppsala, Sweden) according to the manufacturer’s instructions. Briefly, 25 μl of each sample, control replicate, and calibrator was pipetted into the designated wells, followed by the addition of 100 μl of 1× enzyme conjugate solution. The plate was incubated on a shaker at room temperature for 1 h and then washed 5 times with 350 μl of 1× wash buffer. Subsequently, 200 μl of 3,3′,5,5′-tetramethylbenzidine (TMB) substrate was added to each well and incubated for 15 min at room temperature, after which 50 μl of Stop Solution was added. Absorbance was measured at 450 nm using the microplate reader. Insulin concentrations were determined by plotting the standard curve, and the stimulation index was determined as the ratio of insulin secretion under stimulatory versus basal glucose conditions.

### Gene expression analysis

Following 24 h of treatment with 25 μg/ml EVs, total RNA was extracted from EndoC-βH1 spheroids and human islets using Trizol reagent (Thermo Fisher Scientific), and reverse transcribed into cDNA using the High-Capacity cDNA Reverse Transcription Kit (Thermo Fisher Scientific) according to the manufacturer’s instructions. For each condition, *n* = 3 samples were analyzed, with each sample consisting of either 100 spheroids or 100 human islets independently treated. qPCR was performed using Luna Universal qPCR Master Mix (New England Biolabs, Ipswich, MA, USA) on a CFX Opus 96 Real-Time PCR System (Bio-Rad). Glyceraldehyde 3-phosphate dehydrogenase (GAPDH) was used as a control housekeeping gene, and primers were ordered from Integrated DNA Technologies (Coralville, IA, USA) (Table 1).

**Table 1.** Primer pairs used for qPCR analysis obtained from ref. [24]

Gene	Forward primer sequence (5′-3′)	Reverse primer sequence (5′-3′)
INS	GAA CGA GGC TTC TTC TAC AC	ACA ATG CCA CGC TTC TG
PDX1	ATG GAT GAA GTC TAC CAA AGC	CGT GAG ATG TAC TTG TTG AAT AG
SUR1	CGA TGC CAT CAT CAC AGA AG	CTG AGC AGC TTC TCT GGC TT
SLC2A2	TGC TCA CAT AAC TCA TCC AAG	TCA CTG CTG TCT CTG TAT TCC
GCG	ACC AGA AGA CAG CAG AAA TG	GAA TGT GCC CTG TGA ATG
GAPDH	CAC CCA CTC CTC CAC CTT TG	CCA CCA CCC TGT TGC TGT AG

### Protein identification in EndoC-βH1 spheroids and human islets after EV exposure

Proteins from EV-treated and untreated EndoC-βH1 spheroids and human islets were identified using liquid chromatography–tandem mass spectrometry (LC-MS/MS). For each proteomic sample, we pooled 100 EndoC-βH1 spheroids or 100 human islets per condition prior to protein extraction to ensure sufficient protein yield. Lipids and other contaminants were removed by loading the proteins onto a single stacking gel band, which was reduced with dithiothreitol, alkylated with iodoacetic acid, and digested with trypsin. Extracted peptides were resolubilized in 0.1% aqueous formic acid and loaded onto an Acclaim PepMap (75 μM ID × 2 cm C18 3 μM beads) (Thermo Fisher Scientific) precolumn and then onto an Acclaim PepMap EASY-Spray (75 μM × 15 cm with 2 μM C18 beads) (Thermo Fisher Scientific) analytical column for separation using a Dionex Ultimate 3000 uHPLC (Thermo Fisher Scientific) at 250 nl/min with a gradient of 2% to 35% organic (0.1% formic acid in acetonitrile) over 3 h. Peptide analysis was performed using an Orbitrap Fusion mass spectrometer (Thermo Fisher Scientific) at a resolution of 120,000 [full width at half-maximum (FWHM) in MS1] with HCD sequencing (15,000 resolution) at top speed for all peptides with a charge of 2+ or greater. The raw data were converted to Mascot Generic Format (*.mgf) and analyzed with the Mascot 2.6.2 search engine against the UniProt 2023 human protein database. The results were then processed and visualized using Scaffold Q+S software version 5.0.1 (Proteome Software, Portland, OR, USA).

### Bioinformatic analysis

The initial step in the data clean-up process involved the removal of common contaminants and cRAP-listed proteins (Supplementary Excel file). The data were then merged to identify differentially expressed proteins (DEPs) and proteins specific to EV-treated and untreated samples. Fold changes in DEPs were quantified by comparing EV-treated samples to untreated samples following a previously established protocol [23]. A 2-fold change threshold was used to classify proteins as up-regulated (>2-fold) or down-regulated (<0.5-fold). Analysis was based on total spectral counts of proteins identified via LC-MS/MS. Gene ontology (GO) terms for identified proteins were also assessed. The top 10 annotations for biological processes were determined using the STRING database version 12.0. The terms were merged using the database’s analysis function based on a similarity score ≥ 0.1.

### Statistical analysis

Results were obtained from 3 independent biological replicates. Differences were considered statistically significant at a *P* value of <0.05. Data are presented as the mean ± standard error. Statistical differences were evaluated using Welch’s *t* test, one-way analysis of variance (ANOVA), or 2-way ANOVA, with a 95% confidence level. All statistical analyses were performed using the GraphPad Prism software version 10.2.0.

## Results

### EV isolation and characterization

**Fig. 1.**
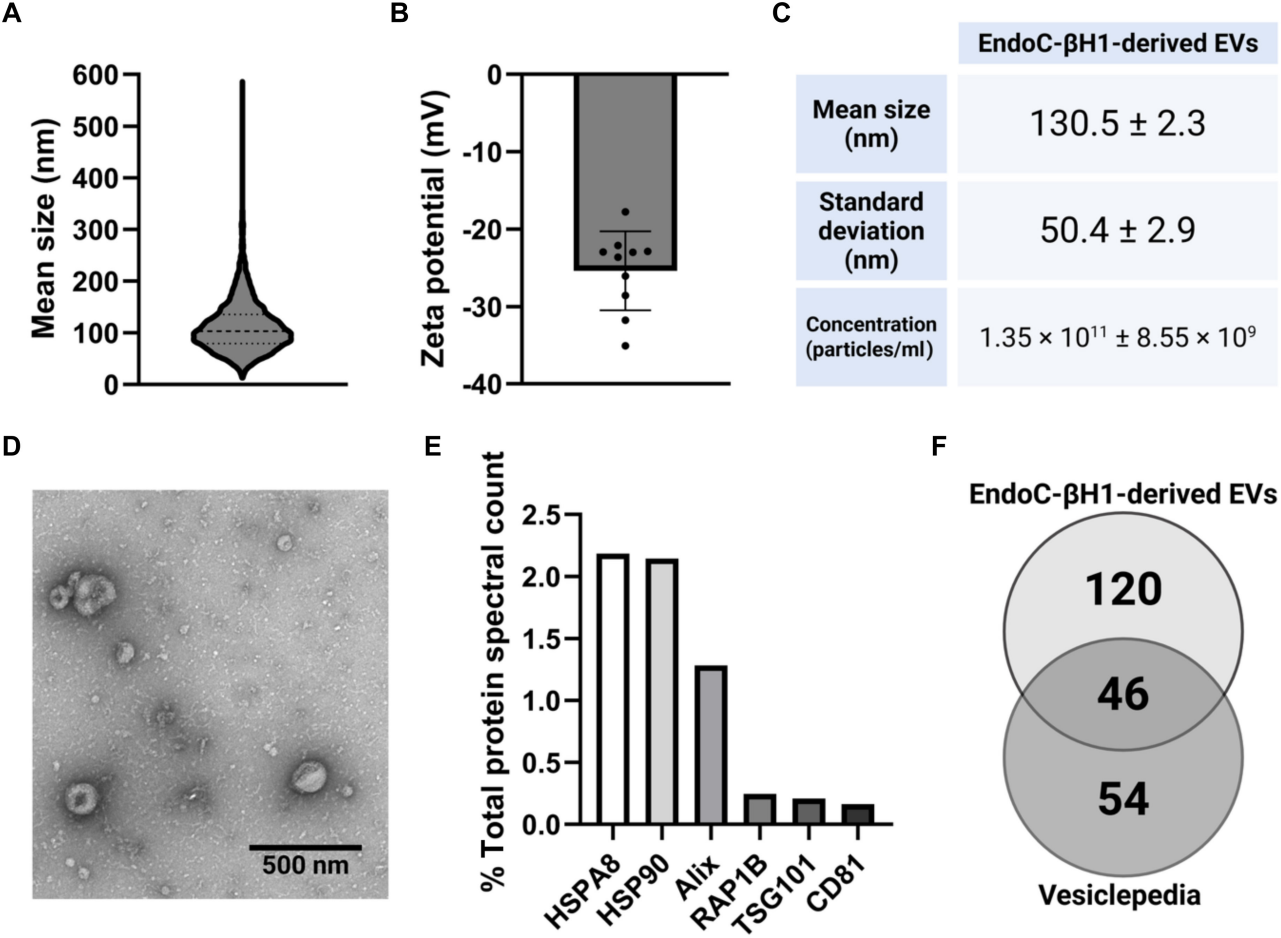
Characterization of extracellular vesicles (EVs). Nanoparticles isolated from EndoC-βH1 human cell line showed characteristics common to EVs. (A) Size distribution pattern. (B) Average zeta potential from 10 repeat reads. (C) Mean particle size, standard deviation, and particle concentration obtained using NTA. (D) Representative transmission electron microscopy (TEM) image of isolated particles. Scale bar, 500 nm. (E) Percent of total spectral counts of common EV markers detected by liquid chromatography–tandem mass spectrometry (LC-MS/MS) in EVs. (F) Venn diagram showing protein overlap between the proteomic profile of the isolated EVs and the top 100 Vesiclepedia proteins.

EVs could be successfully isolated from EndoC-βH1 using ultracentrifugation. The conditioned media (400 ml) yielded ~350 μg of total EV proteins. The EVs had a mean hydrodynamic size of 130.5 ± 2.3 nm and were colloidally stable with a mean surface charge of −28 ± 3.5 mV (Fig. 1A to C). Characteristic EV morphology was observed through TEM (Fig. 1D), while Western blotting further validated the identity of the isolated particles by demonstrating the expression of the canonical EV marker TSG101 (Fig. S1).

Proteomic analysis of the EVs further confirmed their vesicular identity. Canonical markers were detected by MS, including CD81, TSG101, Alix, and HSPA8 (Fig. 1E). Moreover, a total of 166 proteins were identified within the EV preparations, of which 46 overlapped with the top 100 most frequently reported EV proteins curated in the Vesiclepedia database, indicating alignment with commonly reported EV protein compositions (Fig. 1F) [24,25].

### EVs do not present cytotoxic effects in spheroids and islets

**Fig. 2.**
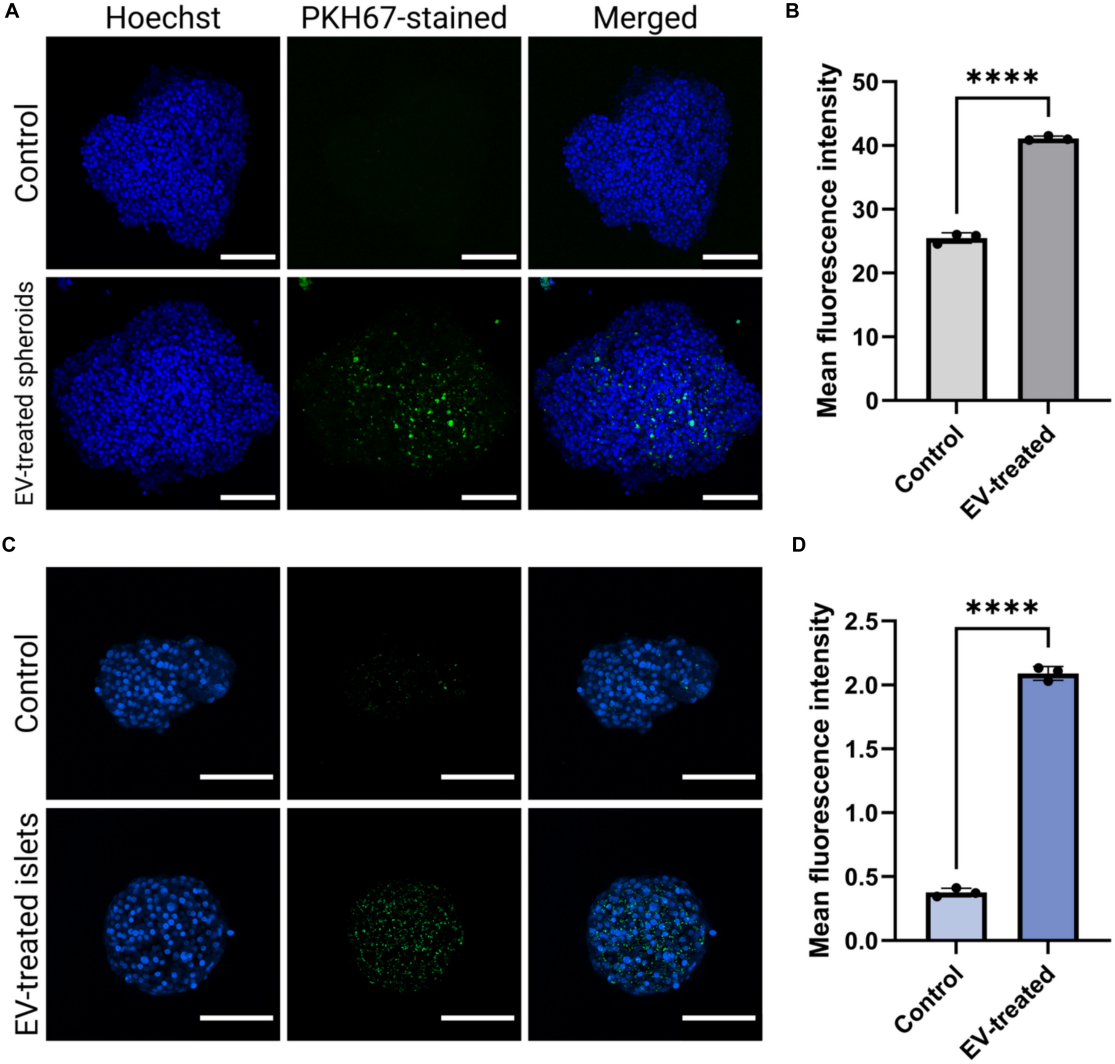
Uptake assessment of EVs. Spheroids and islets can readily internalize EVs. (A) Confocal microscopy imaging of Hoechst-stained spheroids (blue) after 24-h incubation with PKH67-stained EVs (green). Scale bars, 100 μm. (B) Semiquantitative analysis of mean fluorescence intensity of EV-treated versus nontreated control spheroids (*n* = 3 independently treated and stained spheroids; ****P < 0.0001). (C) Confocal microscopy imaging of Hoechst-stained islets (blue) after 24-h incubation with PKH67-stained EVs (green). Scale bars, 100 μm. (D) Semiquantitative analysis of mean fluorescence intensity of EV-treated versus nontreated control islets (*n* = independently treated and stained islets; ****P < 0.0001).

The uptake of EVs by spheroids and islets was confirmed by confocal microscopy (Fig. 2). PKH67-labeled EVs (25 μg/ml) were incubated with the spheroids and islets for 24 h, after which a fluorescent signal corresponding to internalized EVs was readily detected (Fig. S2). Compared with untreated controls, EV-treated samples showed markedly higher fluorescence intensities, indicating efficient uptake.

**Fig. 3.**
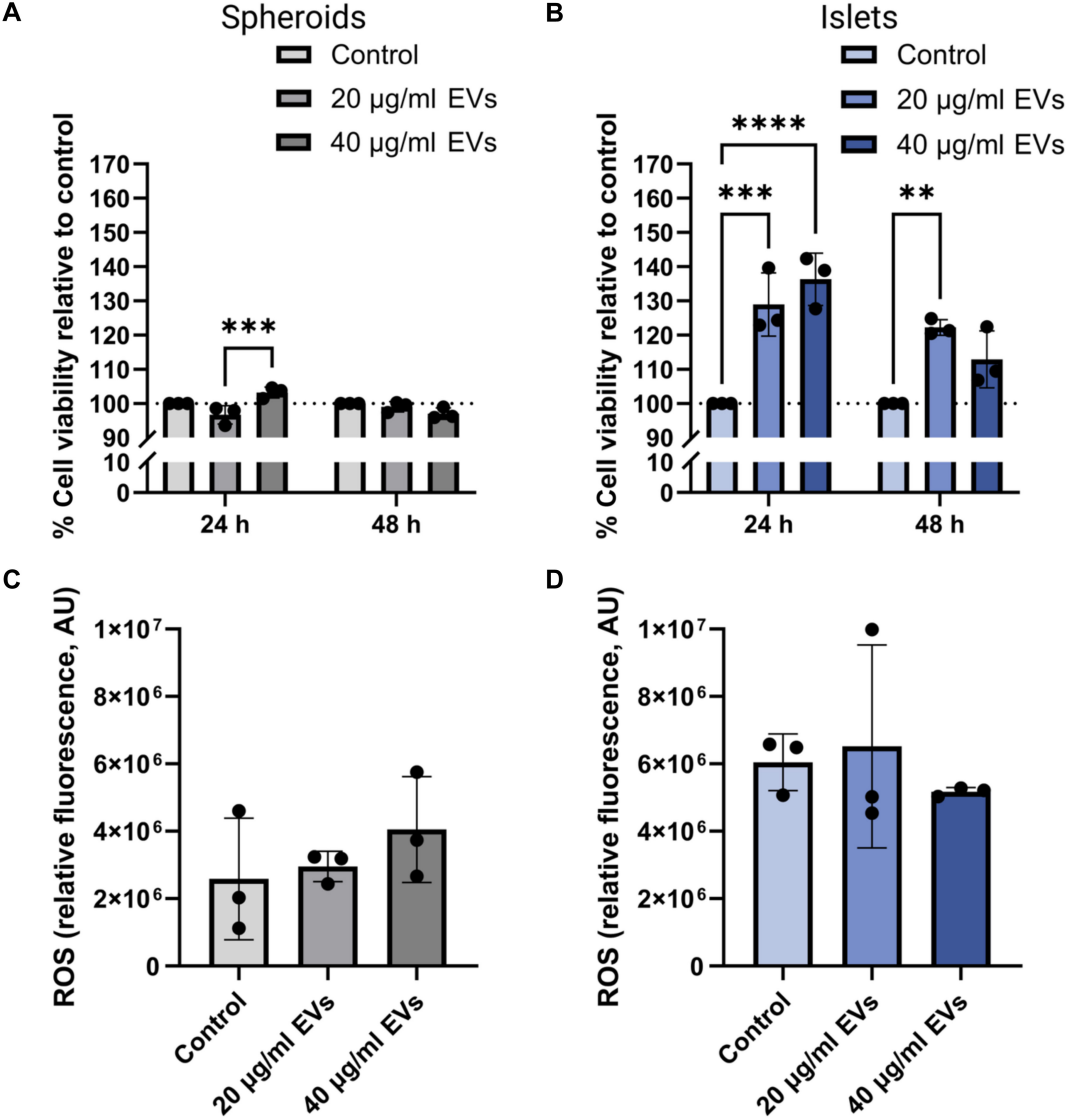
Cytotoxicity assessments. Spheroids and islets were treated with EVs, and metabolic activity was assessed over time, showing no detectable cytotoxic effects. (A and B) Percent reduction of alamarBlue relative to the nontreated control groups in spheroids (*n* = 3 samples of 100 independently treated spheroids or islets; ***P < 0.001) and islets (*n* = 3; compared to control: **P < 0.01, ***P < 0.001, and ****P < 0.0001), respectively. (C and D) Reactive oxygen species (ROS) fluorescent measurements after 24 h of EV exposure in spheroids (*n* = 3) and islets (*n* = 3), respectively.

To further evaluate any potential cytotoxic effect of EVs on the cells, metabolic activity was assessed following treatment with 20 or 40 μg/ml EVs for 24 and 48 h. In spheroids, metabolic activity was largely preserved across conditions, with only a modest but significant increase observed at 24 h with 40 μg/ml EVs compared to treatment with 20 μg/ml EVs (*P* < 0.001) (Fig. 3A). No significant difference was observed between the EV-treated spheroids at either dose and the nontreated spheroid control. In contrast, islets showed a more pronounced response, with both EV concentrations significantly enhancing metabolic activity at 24 h compared to the nontreated control, although no significant difference was observed between the 2 doses (Fig. 3B). After 48 h, the significant increase was sustained only in the 20 μg/ml group (*P* < 0.01). These findings suggest that β-cell-derived EVs are well tolerated and may show promise in improving cell health and supporting β-cell functionality, particularly in islets. Similarly, oxidative stress, measured by ROS fluorescence intensity at 535 nm, remained unchanged in both spheroids (Fig. 3C) and islets (Fig. 3D) compared with untreated controls. No dose-dependent increase was observed, indicating that EV exposure did not elicit a measurable oxidative burden.

### EVs enhance insulin secretion and modestly modulate gene expression in spheroids and islets

**Fig. 4.**
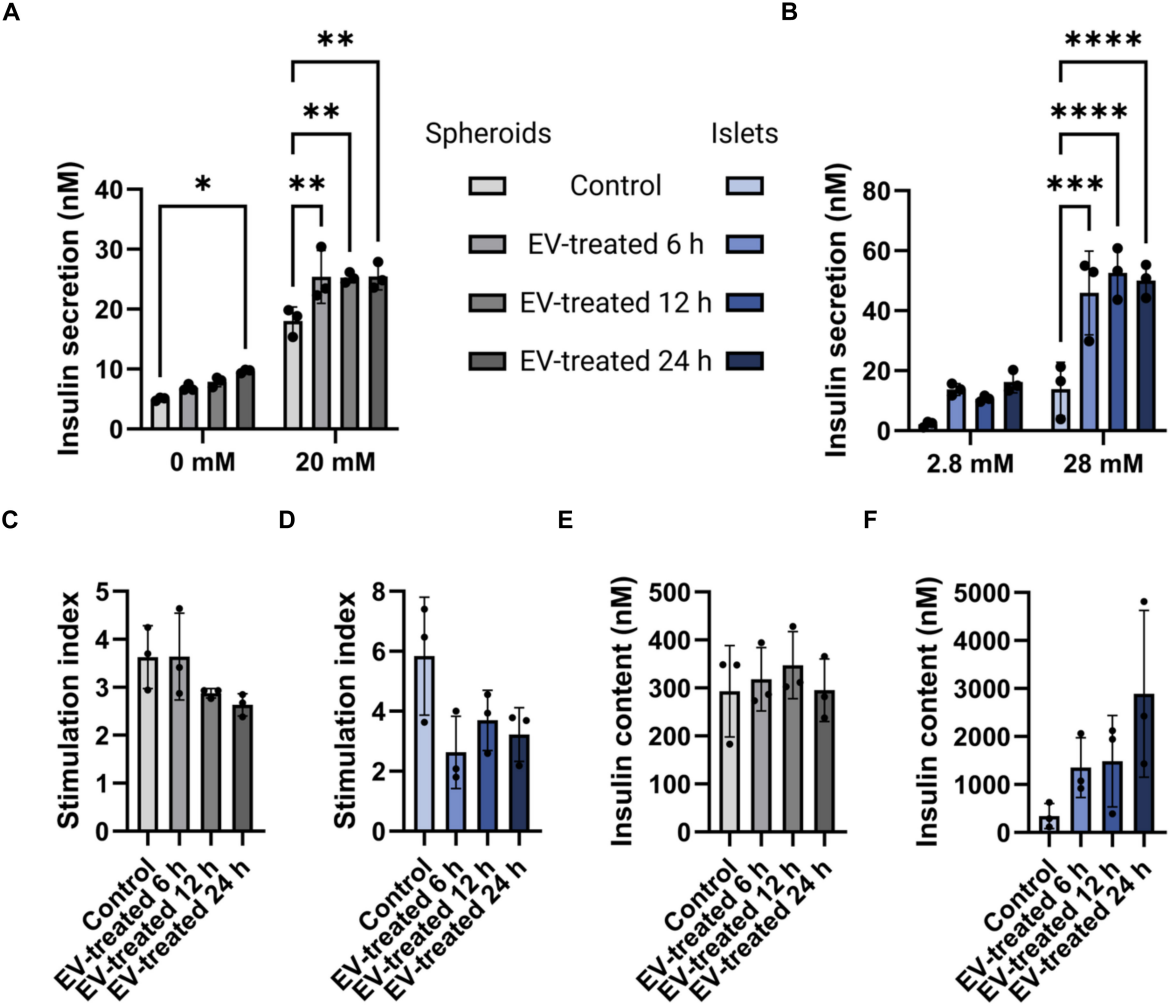
GSIS assay. Spheroids and islets were treated with EVs for different durations to assess functionality. Significantly increased insulin secretion was observed following EV exposure, although it was not dependent on the duration of treatment. (A and B) Insulin concentrations measured by enzyme-linked immunosorbent assay (ELISA) on conditioned media obtained from spheroids (*n* = 3; *P < 0.05 and **P < 0.01) and islets (*n* = 3 samples of 100 independently treated spheroids or islets; ***P < 0.001 and ****P < 0.0001), respectively. (C and D) Stimulation index (high/low glucose ratio) in spheroids (*n* = 3) and islets (*n* = 3), respectively. (E and F) Insulin concentrations measured by ELISA on cell lysates of spheroids (*n* = 3) and islets (*n* = 3), respectively.

The functional impact of EVs on spheroids and islets was evaluated by GSIS assay and subsequent insulin ELISA conducted on both conditioned media and cell lysates. Both spheroids and islets treated with 25 μg/ml EVs for 6, 12, or 24 h exhibited significantly higher insulin secretion and glucose responsiveness at high glucose concentrations compared to untreated controls (Fig. 4A and B). The duration of incubation with EVs, however, did not significantly influence the outcome. Interestingly, in spheroids exposed to EVs for 24 h, insulin secretion was significantly elevated even under basal glucose conditions (0 mM) compared to controls. There was no statistically significant difference in the stimulation index (Fig. 4C and D) nor in intracellular insulin content (Fig. 4E and F) between EV-treated samples compared to the controls. However, an upward trend in insulin content was noticed across the different incubation times in the EV-treated islets compared to the untreated control (Fig. 4F). A complementary surface plasmon resonance assay was also employed to determine the relative insulin concentrations between the control and 24-h EV-treated spheroids and islets. In agreement with the results presented in Fig. 4, it was seen that insulin content was greater than insulin secretion in both spheroids and islets, regardless of whether they were EV treated (Fig. S3A). Additionally, EV-treated spheroids and islets generally had greater detectable insulin compared to their non-EV-treated controls (Fig. S3B).

**Fig. 5.**
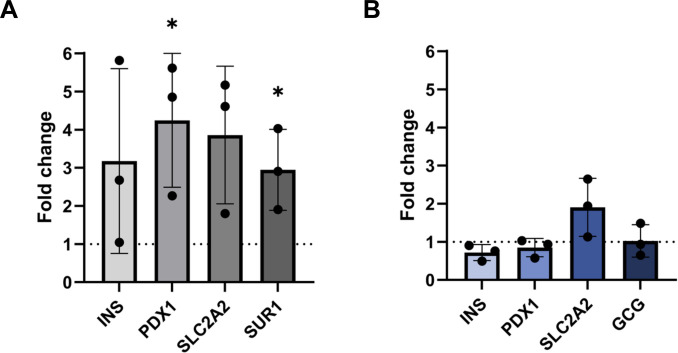
Gene expression analysis. Spheroids and islets were treated with EVs for 24 h, and the effects on the expression levels of relevant genes were assessed, demonstrating a more pronounced effect in spheroids than in islets. (A) Fold change in expression levels of spheroid-relevant genes compared to nontreated control (*n* = 3 samples of 100 independently treated spheroids or islets; compared to control: *P < 0.05). (B) Fold change in expression levels of islet-relevant genes compared to nontreated control (*n* = 3).

To complement these findings, gene expressions of markers associated with β-cell growth and function, such as INS, PDX1, SUR1, and SLC2A2, were examined after 24 h of EV exposure, and the fold changes were compared to those in nontreated cells. For islets, glucagon (GCG) expression was analyzed instead of SUR1, as human islets contain α-cells that secrete glucagon, whereas the spheroids are composed exclusively of β-cells. As shown in Fig. 5A, the expression levels of PDX1 and SUR1 were significantly increased in EV-treated spheroids, while INS and SLC2A2 showed a nonsignificant upward trend. Interestingly, EV treatment had little effect on gene expression in the islet samples (Fig. 5B), except for SLC2A2, which exhibited an almost 2-fold increase in expression compared to the control, although this change did not reach statistical significance.

### EVs reprogram proteomic profiles to support metabolism, vesicle trafficking, and insulin secretion in spheroids and islets

**Fig. 6.**
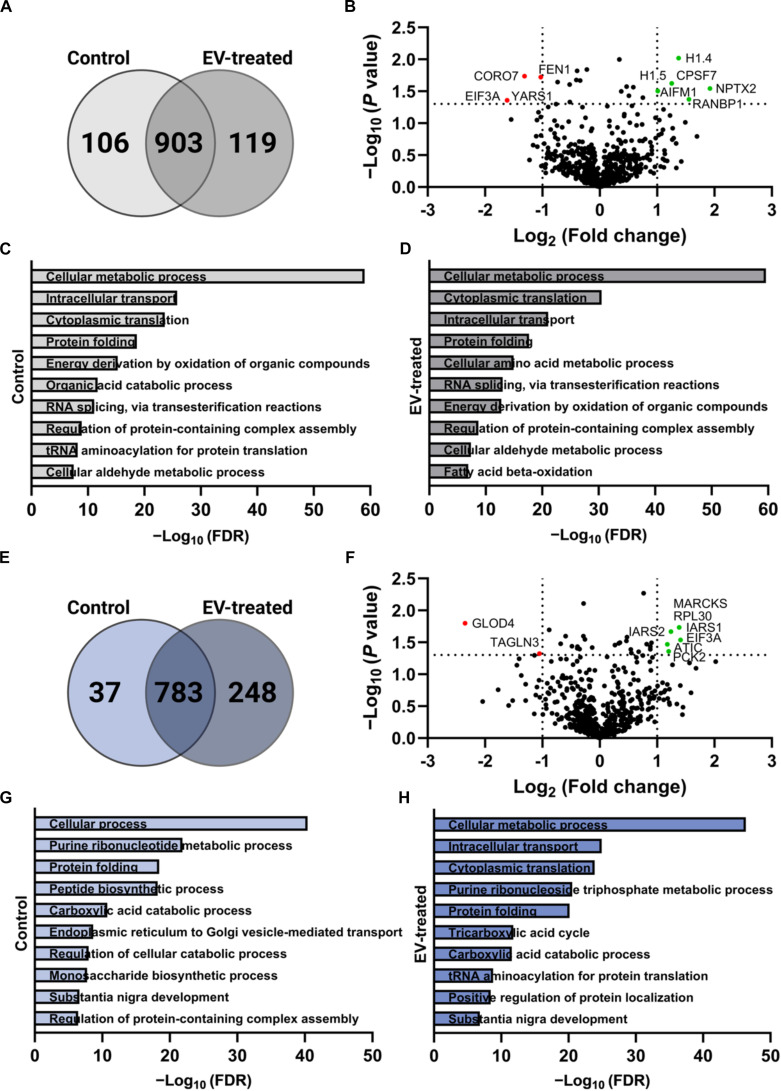
Bioinformatic characterization of EV-treated spheroids and islets. The proteomic profiles of the spheroids and islets were determined by MS after 24 h of EV exposure. Bioinformatic analysis demonstrated a shift toward profiles more conducive to enhanced β-cell functionality. (A) Venn diagram of protein overlaps in spheroid samples. (B) Volcano plot showing fold change in the levels of differentially expressed proteins (DEPs) in EV-treated spheroids versus nontreated control. Colored data points represent proteins with significant differential expressions (horizontal line: *P* < 0.05) that exceed a 2-fold change threshold (vertical lines: up-regulation > 2 or down-regulation < 0.5). (C and D) Merged gene ontology (GO) analysis for the top 10 biological process terms in nontreated control and EV-treated spheroids, respectively, based on false discovery rates (FDRs). (E) Venn diagram of protein overlaps in human islet samples. (F) Volcano plot showing fold change in the levels of DEPs in EV-treated islets versus nontreated control. Colored data points represent proteins with significant differential expressions (horizontal line: *P* < 0.05) that exceed a 2-fold change threshold (vertical lines: up-regulation > 2 or down-regulation < 0.5). (G and H) Merged GO analysis for the top 10 biological process terms in nontreated control and EV-treated human islets, respectively, based on FDR.

To identify potential drivers underlying the functional effects of EVs, proteomic profiles of EV-treated spheroids and islets were generated via bioinformatic analysis and compared with untreated controls. In spheroids, EV treatment modestly increased the total number of detected proteins (1,022 versus 1,009 in untreated control). A total of 903 proteins were differentially expressed, including 119 unique to EV-treated spheroids and 106 unique to the control group (Fig. 6A). Relative to the control group, EV treatment significantly up-regulated proteins relevant to gene expression regulation and vesicle trafficking, such as H1.4 and NPTX2, while down-regulated proteins included those involved in DNA replication and cell proliferation (Fig. 6B). Additionally, the top 5 proteins unique to the EV-treated spheroids with the highest spectral counts were TUBB4A, RAB1B, SCG2, HMGB2, and FLNB, all playing key roles in cytoskeletal organization, vesicle trafficking, and secretory granule formation (Table 2). These patterns demonstrate a shift away from general proliferative and biosynthetic activity toward a more metabolically active and functionally specialized state in EV-treated β-cell spheroids [26].

**Table 2.** Top 5 unique protein counts (→) and up-regulated (↑) or down-regulated (↓) DEPs

Protein identifier	Expression	Common role ^a^
**In EV-treated versus untreated β-cell-derived spheroids**		
TUBB4A	**→**	Major component of the cellular cytoskeletal system
RAB1B	**→**	Involved in the regulation of intracellular trafficking
SCG2	**→**	Involved in the regulation of secretory granule biogenesis
HMGB2	**→**	Plays various roles in transcription regulation, antimicrobial activity, and chemokine activity that promotes the migration and proliferation of endothelial cells
FLNB	**→**	Plays a role in transmembrane anchoring
H1.4 and H1.5	**↑**	Involved in the regulation of gene transcription
CPSF7	**↑**	Plays a role in mRNA maturation
NPTX2	**↑**	Plays a role in the regulation of cell plasticity and cell-to-cell communication
RANBP1	**↑**	Involved in RAN-dependent nucleocytoplasmic transport
CORO7	**↓**	Involved in post-Golgi transport
FEN1	**↓**	Plays a role in DNA cleavage and repair
EIF3A	**↓**	Involved in the translation of mRNAs relevant to cell proliferation and differentiation
YARS1	**↓**	Catalyzes the attachment of tyrosine to tRNAs and regulates poly-ADP-ribosylation
**In EV-treated versus untreated human islets**		
TUBB2B	**→**	Major component of the cellular cytoskeletal system
FASN	**→**	Key player in the biosynthesis of long-chain fatty acids
SUCLG1	**→**	Involved in cellular metabolism and energy production
RAB6A	**→**	Involved in the regulation of intracellular trafficking
GNB2	**→**	Involved in the modulation of transmembrane signaling
MARCKS	**↑**	Plays a role in cell adhesion, motility, phagocytosis, and exocytosis
RPL30	**↑**	Ribosomal component
IARS	**↑**	Catalyzes the attachment of isoleucine to tRNAs
EIF3A	**↑**	Component of the eukaryotic translation initiation factor 3 that is involved in the translation of mRNAs relevant to cell proliferation and differentiation
ATIC	**↑**	Involved in purine biosynthesis and insulin receptor autophosphorylation and internalization
PCK2	**↑**	Plays important roles in glyceroneogenesis and gluconeogenesis
GLOD4	**↓**	Involved in the regulation of cell growth
TAGLN3	**↓**	Plays a role in the regulation of cellular cytoskeletal system

^a^ Protein functions were obtained from the UniProt database (accessed on 2025 October 1).

Moreover, while the top 10 GO terms for biological processes pointed to an overall conservation among core β-cell functions between the 2 groups, the control spheroids were enriched in organic acid catabolic process and tRNA aminoacylation for protein translation (Fig. 6C), whereas the EV-treated spheroids were enriched in cellular amino acid metabolic process and fatty acid β-oxidation (Fig. 6D). These shifts further support that EV treatment promotes metabolic reprogramming consistent with improved β-cell activity.

Similarly, EV-treated islets also showed a higher number of proteins compared to the control group (1,031 versus 820). Among these, 783 proteins were shared between groups, with 248 unique to EV-treated islets and 37 unique to controls (Fig. 6E). Differential expression analysis further showed that the EV-treated islets displayed significant up-regulation in proteins relevant to translation, metabolic activity, vesicle trafficking, and exocytosis (Fig. 6F). Similar roles were observed among the top 5 proteins uniquely identified in the EV-treated group, corroborating the positive effect of β-cell EVs on enhancing protein synthesis, energy metabolism, and insulin secretion capacity in human islets (Table 2). Additionally, GO analysis again revealed a positive shift toward processes associated with functional enhancement following EV treatment (Fig. 6G and H).

No significant enrichment of canonical Wnt signaling components or exosome-associated Rab guanosine triphosphatases (GTPases) (Rab6, Rab7, Rab10, Rab11, Rab14, Rab16) was observed in EV-treated spheroids; instead, proteomic changes were dominated by proteins involved in vesicle trafficking, cytoskeletal remodeling, metabolism, and β-cell secretory function.

## Discussion

Research characterizing β-cell-derived EVs and evaluating their effect on primary human islets remains limited. In the present study, we directly compared the effect of β-cell-derived EVs on β-cell spheroids and human donor islets by examining key functional parameters. We found that EVs are efficiently internalized without impairing the metabolic activity and have the potential to significantly enhance insulin secretion in response to high glucose levels. Human islets also showed increased metabolic activity after EV treatment, consistent with reports that EVs mediate metabolic reprogramming across islet cell types, including α- and γ-cells, which may explain the absence of this effect in the β-cell spheroids [27,28]. Regarding dose selection, the EV concentrations used for the GSIS assessment were informed by preliminary exploratory titrations performed to identify a practical range that produced measurable functional responses without inducing cytotoxicity. These pilot tests indicated that effects begin to emerge around this concentration threshold, supporting its use for subsequent experiments. Because these optimizations were exploratory and not intended as a formal dose–response study, we focus here on the comparative behavior of spheroids and islets under standardized treatment conditions while acknowledging that future work will need to define precise dose–response relationships and temporal dynamics using more refined quantitative approaches.

Our assays revealed no significant changes in ROS levels following EV treatment, neither in spheroids nor in islets. Instead, the primary effects emerged at the transcriptional level. In spheroids, EVs up-regulated PDX1 and SUR1, markers central to β-cell function and insulin secretion. PDX1 is a transcription factor that plays a critical role in β-cell development, function, and survival, while SUR1 modulates insulin release via K(ATP) channel activity [29]. We also observed upward trends in INS and SLC2A2 expression, suggesting enhanced insulin synthesis and glucose responsiveness [30]. Taken together, these findings suggest that human β-cell-derived EVs may enhance insulin production by improving the expression of key functional genes in β-cell spheroids.

In contrast, gene expression changes in primary islets were minimal, likely reflecting the lower responsiveness of primary cells compared with immortalized EndoC-βH1 cells [20,31]. Nevertheless, intracellular insulin content showed an upward trend in EV-treated islets after 24 h, suggesting that longer exposure may reveal time-dependent effects. Future studies should assess multiple time points to further elucidate the relationship between EV treatment and insulin synthesis. The consistent increase in GSIS indicates that additional mechanisms, independent of transcriptional changes, may be driving the response. One possibility is the direct transfer of insulin or preproinsulin cargo via EVs, as suggested by recent work demonstrating preproinsulin on islet EV [32,33]. Another possibility is the contribution of EV-associated miRNAs in regulating β-cell function. miRNAs are known to be abundant in EVs and play important roles in regulating protein expression [34]. For instance, miR-212/213 released by β-cells has been shown to promote and regulate insulin secretion and drive induced pluripotent stem cell (iPSC) differentiation into insulin-producing cells [18]. Similarly, β-cell-derived EVs containing miR-29 have also been reported to regulate glucose sensitivity, further supporting the observed increasing trend in SLC2A2 gene expression after EV treatment in both spheroids and islets. Since insulin production in β-cells is an SLC2A2-mediated process triggered by high extracellular glucose, this observation reinforces the potential role of EV-associated miRNAs in enhancing insulin secretion [35].

Bioinformatic analysis provided further support for these functional changes, with increased abundance of proteins involved in key pathways. In EV-treated spheroids, several of the most up-regulated proteins play important roles in β-cell functionality. For instance, H1.4 and H1.5, which regulate gene transcription, are highly up-regulated. H1 proteins protect the DNA from double-strand breaks by limiting access of damaging factors, while H1.5 specifically promotes chromatin compaction, further stabilizing the genome [36].

Moreover, CPSF7, a protein responsible for the cleavage and polyadenylation of pre-mRNAs and thus regulating RNA metabolism, was among the top up-regulated DEPs in EV-treated spheroids [37]. While no direct link to diabetes or β-cells has been established, RNA metabolism regulation is critical for maintaining cellular function. RANBP1, a RAN binding protein essential for RNA and protein transport in the nucleus, was also identified and is known to play a key role in controlling autophagy and promoting cell survival under hyperglycemic conditions [38].

Interestingly, most highly expressed proteins unique to EV-treated spheroids are involved in intracellular trafficking. For instance, TUBB41 is a key cytoskeleton protein that mediates the movement of intracellular components. RAB1B and SCG2, both up-regulated in EV-treated spheroids, regulate exocytosis of insulin granules via the protein kinase A (PKA) signaling pathway, facilitating insulin granule mobilization and exocytosis [26,27]. However, when it comes to canonical Wnt signaling, no other Rab GTPases were among the significantly altered proteins in the EV-treated spheroids and were not uniquely enriched relative to controls. This is consistent with the broader pattern that EV exposure primarily enhanced proteins linked to vesicle trafficking, cytoskeletal organization, metabolic reprogramming, and β-cell secretory function, rather than uniformly increasing the classical multivesicular body/EV-associated Rab GTPase cohort.

Similar outcomes were observed in human islets, where most up-regulated DEPs support cellular function and survival. These are IARS and EIF3A, both involved in regulating RNA metabolism and protein synthesis [39]. EIF3A plays a dual role in translationally regulating glucose metabolism through an eIF3a–Rheb–AMPK signaling pathway and modulating the function of B lymphocytes [40]. In addition, overexpressed MARCKS proteins, which are known mediators of vesicle trafficking, were identified [41]. Other highly expressed DEPs include ATIC enzymes, which are involved in purine biosynthesis and regulate insulin internalization through insulin receptor phosphorylation [42]. Finally, PCK2, another highly expressed DEP, plays a complex role in diabetes and cell metabolism. Specifically, it regulates cell survival under amino acid starvation and endoplasmic reticulum (ER) stress. In T1D, the adaptive ER stress response in β-cells is often highly dysregulated. Therefore, increased PCK2 expression may enhance survival against ER stress and lead to improved β-cell function [43]. In addition, TUBB2B, a cytoskeleton protein involved in exocytosis processes, was among the most up-regulated proteins unique to EV-treated islets. SUCLG1, a mitochondrial enzyme from the phosphatase family, was also highly up-regulated in EV-treated islets. Its role in maintaining cell metabolic activity supports the increased metabolic activity observed in these islets, as measured by the alamarBlue assay [44]. Consistent with our previous findings, proteins involved in the release of insulin granules were among the most up-regulated in response to glucose concentration shifts, specifically RAB6A and GNB2 [45,46]. Additionally, FASN, a fatty acid synthase uniquely up-regulated in EV-treated islets, plays a significant role in suppressing immune responses toward pancreatic islets [47].

Proteomic profiling of the EVs revealed enrichment of proteins linked to β-cell regulated secretion, including insulin and secretogranin-2 (SCG2), a dense-core granule protein and source of bioactive peptides in endocrine cells. Previous studies have shown that β-cells release EVs containing intracellular and secretory pathway components, supporting a granule-linked origin for a subset of EV cargo. Moreover, the detection of fibronectin suggests a potential role for ECM–integrin interactions, which are known to spatially organize and potentiate insulin secretion in β-cells. These proteomic patterns support a model in which EV exposure enhances vesicle trafficking and β-cell functional specialization rather than activating canonical Wnt signaling [48].

Collectively, these proteomic patterns support a model in which EV exposure enhances vesicle trafficking and β-cell functional specialization rather than activating canonical Wnt signaling. Because this is an exploratory discovery-proteomics study, these associations should be viewed as hypothesis-generating; orthogonal validation (e.g., targeted proteomics or immunoblotting of RAB1B/SCG2/TUBB4A and functional insulin secretion assays) will be essential to confirm directionality and magnitude of the observed changes across additional donors and islets and spheroid preparations.

In terms of EV characterization, although our Western blot analysis included only TSG101, our proteomic and bioinformatic profiling of the EV preparations identified a broad range of established EV markers consistent with MISEV2018 recommendations, supporting the purity of the isolates.

Despite the uncovered potential of β-cell-derived EVs as therapeutic tools to improve insulin production in 2 independent human β-cell models, the translational potential of these EVs and the feasibility of producing them in compliance with clinical standards remains an unavoidable obstacle. For any clinical application, EVs would need to be generated under Good Manufacturing Practice (GMP) conditions using standardized, scalable, and quality-controlled processes. In this context, 2 realistic producer sources can be envisioned: primary human β-cells/islets, which best mimic native physiology but are limited by scarcity and donor variability, and immortalized human β-cell lines such as EndoC-βH1, which offer a stable, renewable, and more GMP-compatible platform. Several ongoing EV-based therapeutic programs rely on immortalized or stem-cell-derived cell lines, underscoring the feasibility of this approach [49,50]. Another important limitation of this study relates to the use of human islets from a single donor. Although the donor presented with obese class I BMI, no metabolic or systemic complications were reported, and the islets exhibited normal HbA1c (5.9%), high purity (90%), and high viability (95%), indicating preserved metabolic function at isolation. Nonetheless, donor-to-donor variability remains a significant challenge in islet research, and future studies incorporating multiple independent donors will be essential to capture inter-individual differences. Similarly, the number of biological and technical replicates was constrained by the limited availability and high cost of primary human islets, as well as the substantial material requirements needed to perform parallel GSIS, ELISA, gene expression, and proteomic analyses. To partially mitigate this constraint, we complemented the islet experiments with an independent β-cell model (EndoC-βH1 spheroids) and validated outcomes across multiple orthogonal functional readouts. Even with these limitations, the consistency of the observed effects across 2 human β-cell systems supports the robustness of our findings while underscoring the need for larger, multi-donor follow-up studies.

Based on the functional enhancements observed in our data, we envision β-cell-derived EVs to be used adjunctively with clinical islet transplantation in patients with T1D. Because islet grafts experience substantial early functional loss due to ischemia and delayed revascularization, EVs could theoretically be administered into the portal vein together with donor islets, mirroring current transplantation procedures, to support early graft survival and functional integration.

However, we fully acknowledge that our study does not establish superiority of β-cell-derived EVs over other clinically advanced EV sources such as MSC-derived EVs, which are easier to scale and isolate. A head-to-head comparative study under controlled conditions will be required to determine which EV source provides the greatest benefit for β-cell support. Likewise, demonstrating clinical compatibility will require additional mechanistic, functional, and manufacturing-oriented studies.

Together, these data show that β-cell-derived EVs enhance insulin secretion and support β-cell survival in both immortalized β-cell line-derived spheroid and primary pancreatic β-cells. The parallel outcomes in the 2 models validate EndoC-βH1 spheroids as a relevant system for studying islet biology and highlight β-cell EVs as promising candidates for EV-based therapies in T1D. While these findings highlight β-cell EVs as promising exploratory candidates for therapeutic strategies in T1D, further mechanistic validation, comparative studies with other EV sources, and GMP-compatible production approaches will be essential before any clinical application can be envisioned.

## Conclusion

This proof-of-concept study provides compelling evidence that β-cell-derived EVs positively influence β-cell function and survival, in both immortalized β-cell spheroids and primary human islets. Our results demonstrate that EVs are readily internalized without compromising metabolic activity, and importantly, they enhance insulin secretion in response to high glucose concentrations. The observed up-regulation of key functional genes such as PDX1, SUR1, and SLC2A2 in β-cell spheroids, alongside proteomic shifts favoring cellular resilience and intracellular trafficking, supports the notion that EVs modulate β-cell biology at multiple levels. Although these gene expression changes were not as prominent in primary human islets, likely due to their lower susceptibility to external regulation, the consistent trends in insulin secretion and metabolic enhancement indicate that β-cell-derived EVs may exert their effects through additional mechanisms. Notably, the potential direct transfer of insulin precursors or granules within EVs, as well as miRNA-mediated regulation, present promising avenues for further investigation. Our proteomic analyses identified the up-regulation of proteins involved in gene transcription, RNA metabolism, vesicle trafficking, and stress response pathways, all of which converge to support improved β-cell function and survival.

The consistent findings across β-cell spheroids and human islets strengthen the validity of the EndoC-βH1-derived spheroid model for studying human islet biology and for preclinical development of EV-based therapies. Taken together, these insights highlight the therapeutic potential of β-cell-derived EVs as modulators of insulin production and secretion, with implications for innovative treatments in T1D. Future studies focused on elucidating the precise molecular cargo and mechanisms of action of these EVs will be critical to translate these findings into clinical applications.

## Data Availability

The datasets used and/or analyzed in this study are available from the corresponding author on reasonable request.

## Supplementary Materials

Supplementary 1 Figs. S1 to S3 Excel file
