# Supplementary material for: β-Cell-Derived Extracellular Vesicles Boost β-Cell Functionality in Human Pancreatic Islets
Source: Biomater Res. 2026 Mar 17;30:0346. doi: 10.34133/bmr.0346 (PMC12992928; doi:10.34133/bmr.0346)
Supplement: Supplementary 1 — Figs. S1 to S3 Excel file [file bmr.0346.f1.zip › Boucenna et al Supplementary.docx]

**Supplementary Materials for**

**β-cell-derived extracellular vesicles boost β-cell functionality in human pancreatic islets**

Sarah Boucenna^1^, Antoine Karoichan^1^, Michael Yilma Yitayew^2^, John V. L. Nguyen^2^, Maryam Tabrizian^1,2*^

^1^Faculty of Dental Medicine and Oral Health Sciences, McGill University, Montreal, Quebec H3A 1G1, Canada.

^2^Department of Biomedical Engineering, McGill University, Montreal, Quebec H3A 2B4, Canada.

*Address correspondence to: Maryam Tabrizian; [maryam.tabrizian@mcgill.ca](https://mcgill-my.sharepoint.com/personal/AppData/Local/Microsoft/Windows/INetCache/Content.Outlook/ZZI3YJTP/maryam.tabrizian@mcgill.ca)

1. **Detection of EV marker TSG101**


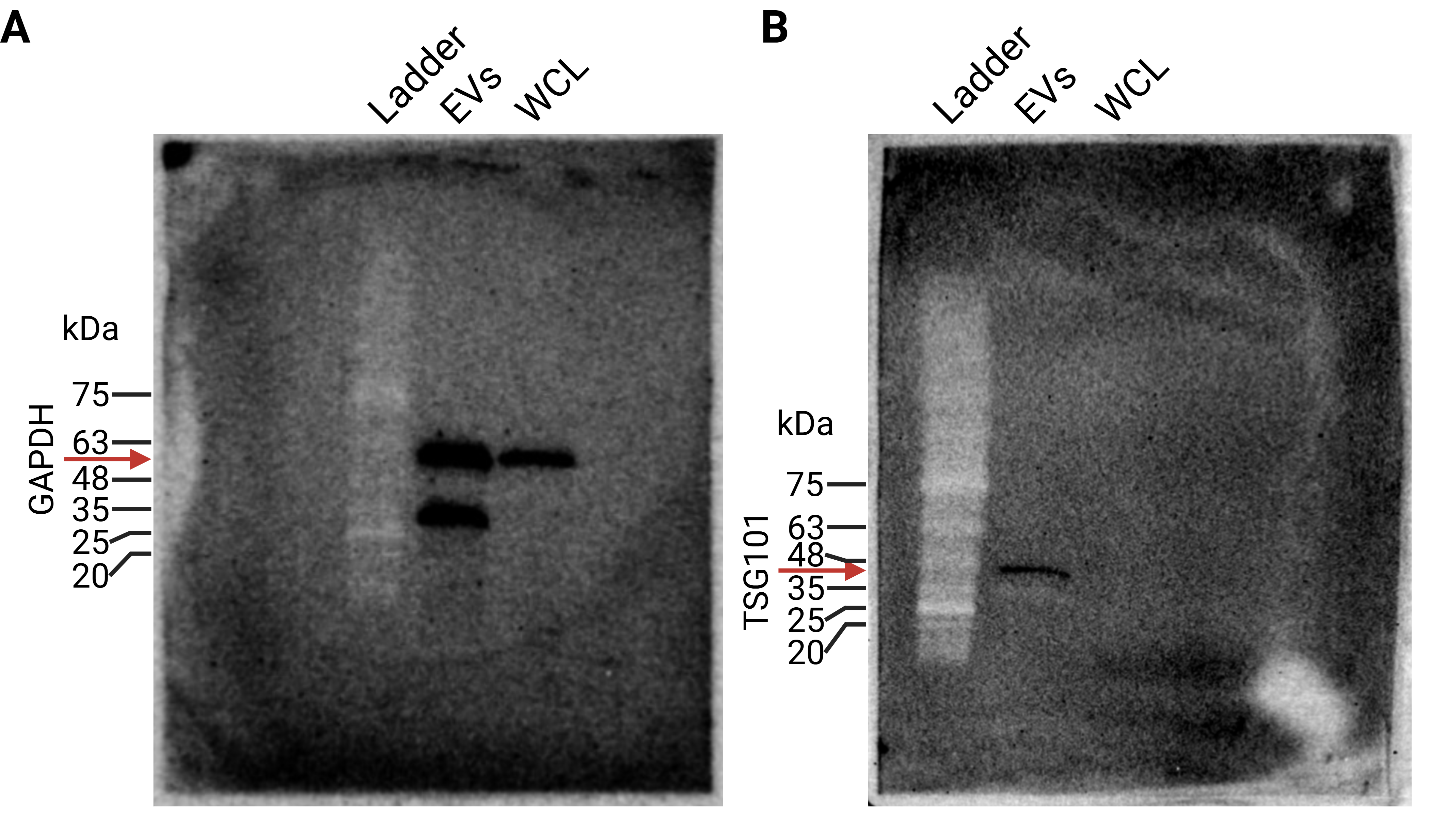


**Fig. S1.** Western blot analysis of (A) GAPDH and (B) TSG101 in extracellular vesicles (EVs) and whole cell lysate (WCL).

1. **Free PKH67 dye control with spheroids**


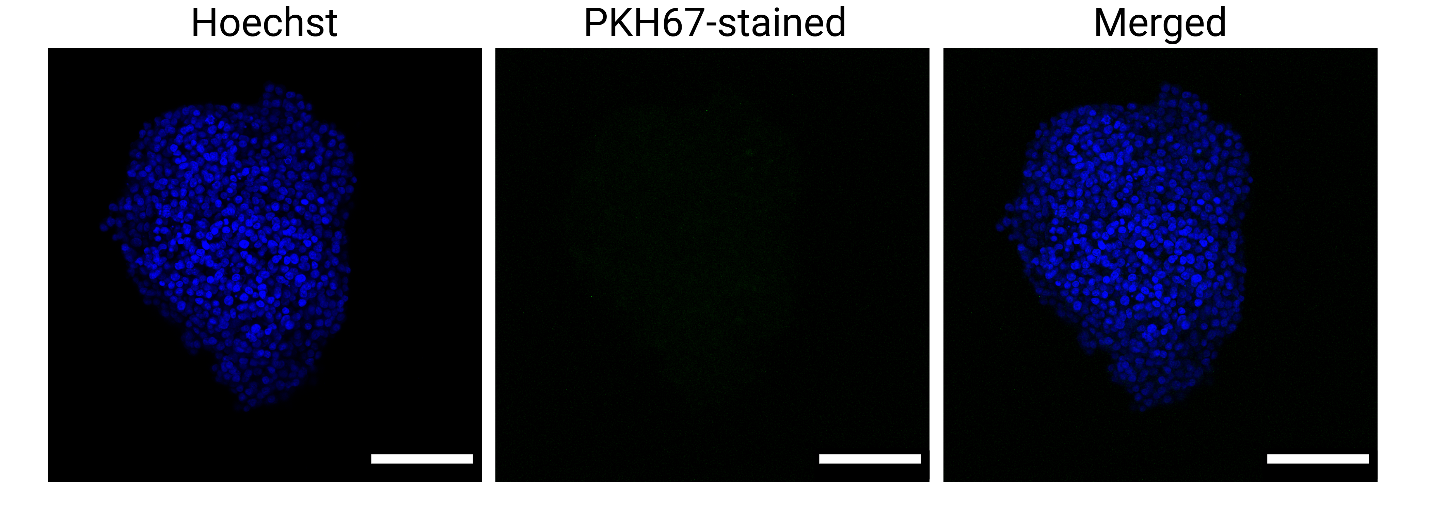


**Fig. S2.** PKH67 in particle-free PBS control added to spheroids. Scale bars = 100 µm.

1. **Surface plasmon resonance for relative insulin determination**

Determination of relative insulin concentrations were conducted using a Sierra SPR-24 Pro (Bruker Daltonics). An SPR High Capacity Amine Sensor (Bruker Daltonics) was activated with a mixture of 400 mM 1-ethyl-3-(3-dimethylaminopropyl)carbodiimide (EDC) and 50 mM N-hydroxysuccinimide (NHS) injected with a flow rate of 5 μL/min for 6 min. Mouse anti-human insulin monoclonal antibody (Prospec-Tany Technogene, Rehovot, Israel) was diluted in sodium acetate buffer pH 5.5 (Bruker Daltonics) and injected with a flow rate of 5 μL/min for 6 min. A 1 M ethanolamine solution was injected at a flow rate of 10 μL/min for 6 min to inactivate the surface. A PBST wash was injected with a flow rate of 10 μL/min for 1 min. For determination of relative insulin concentrations, control and 24 h EV-treated spheroid and islet cell lysate and secretions were injected with a flow rate of 5 μL/min for 6 min. The preconditioning and antibody immobilization were performed with a PBST running buffer. The sensorgram of the ligand-analyte binding is as shown in Fig. S3A. For each injection, the binding signal was quantified as the mean response units (RU) over the final 5 s of the association phase, after bulk refractive index effects had stabilized. These averaged late-association values were plotted as a function of analyte concentration to generate the response-concentration relationship (Fig. 3B). Concentration values were taken from the complementary GSIS assay, as shown in Fig. 4.


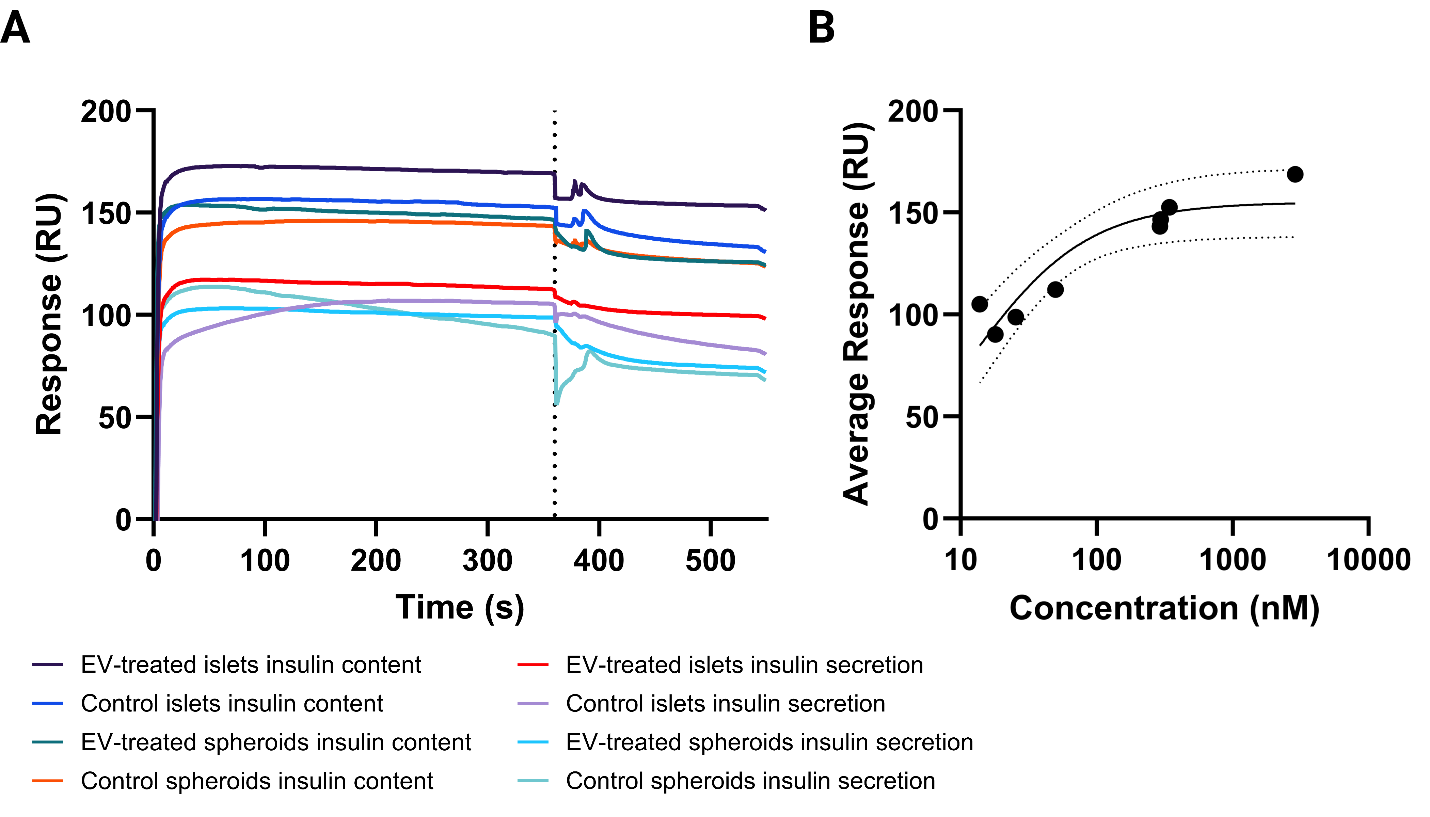


Fig. S3. SPR Insulin-antibody binding assay. (A) SPR sensorgram of association and dissociation of insulin injected over an insulin antibody-immobilized sensor. Dotted line at 360 s indicates end of association phase and start of dissociation phase. (B) Response-concentration relationship derived from late-association SPR signals. These averaged values (points) were plotted as a function of analyte concentration and fit with a steady-state binding model (solid line), with dotted lines indicating the 95% confidence interval of the fit.
